# An *in-situ* infection detection sensor coating for urinary catheters

**DOI:** 10.1016/j.bios.2016.02.059

**Published:** 2016-07-15

**Authors:** Scarlet Milo, Naing Tun Thet, Dan Liu, Jonathan Nzakizwanayo, Brian V. Jones, A. Toby A. Jenkins

**Affiliations:** a Department of Chemistry, University of Bath, Bath BA2 7AY, UK; b School of Pharmacy and Biomolecular Sciences, University of Brighton, BN2 4GJ, UK; c Queen Victoria Hospital NHS Foundation Trust, East Grinstead RH19 3DZ, UK

**Keywords:** Catheter-associated urinary tract infections, *Proteus mirabilis*, Carboxyfluorescein, Hydrogels, Sensor

## Abstract

We describe a novel infection-responsive coating for urinary catheters that provides a clear visual early warning of *Proteus mirabilis* infection and subsequent blockage. The crystalline biofilms of *P. mirabilis* can cause serious complications for patients undergoing long-term bladder catheterisation. Healthy urine is around pH 6, bacterial urease increases urine pH leading to the precipitation of calcium and magnesium deposits from the urine, resulting in dense crystalline biofilms on the catheter surface that blocks urine flow. The coating is a dual layered system in which the lower poly(vinyl alcohol) layer contains the self-quenching dye carboxyfluorescein. This is capped by an upper layer of the pH responsive polymer poly(methyl methacrylate-*co*-methacrylic acid) (Eudragit S100®). Elevation of urinary pH (>pH 7) dissolves the Eudragit layer, releasing the dye to provide a clear visual warning of impending blockage. Evaluation of prototype coatings using a clinically relevant *in vitro* bladder model system demonstrated that coatings provide up to 12 h advanced warning of blockage, and are stable both in the absence of infection, and in the presence of species that do not cause catheter blockage. At the present time, there are no effective methods to control these infections or provide warning of impending catheter blockage.

## Highlights

• Development of a dual-layered diagnostic coating sensor for urinary catheter.
• Provides advance warning of infection by *P. mirabilis* in response to alkaline urine.
• Read out via release of carboxyfluorescein dye from coating into urine leg bag.
• Catheter prototypes ‘report’ infection 12 h before blockage by *P. mirablis.*

## Graphical abstract

**A pH-responsive diagnostic coating for urinary catheters is presented,** to provide advance warning of encrustation and blockage by *P. mirabilis*. Diagnosis is facilitated by the stimulated release of 5(6)-carboxyfluorescein from a hydrogel reservoir in response to increased urinary pH. Catheter prototypes are tested using a clinically relevant continuous flow *in vitro* bladder model system, and artificial urine.

**Figure:**
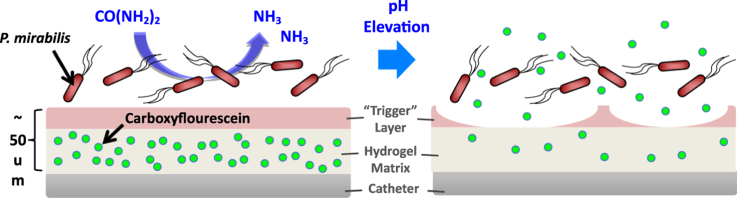

## 1 Introduction

It has been estimated that ~100 million indwelling urinary catheters (IUC) are sold annually worldwide (Saint et al., 2000). In the US alone, ~30 million urinary catheters are fitted each year, making IUCs by far the most commonly deployed medical device, with levels of use far outstripping other common devices such as central venous catheters or fracture fixation devices (Darouiche, 2001). Although in many cases the use of IUCs can benefit patients and greatly aid treatment and recovery, these devices undermine the innate barriers to bacterial colonisation naturally present in the urinary tract, thus predisposing patients to infection by uropathogenic bacteria (Stickler and Zimakoff, 1994; Stickler, 2014). Given the widespread use of these devices, it is perhaps unsurprising that catheter associated urinary tract infections (CAUTIs) are currently among the most common nosocomial infections in many healthcare settings (Stickler, 2014; Jacobsen et al., 2008; Tambyah, 2004; Hooton et al., 2010; Getliffe and Newton, 2006). CAUTIs pose a serious risk to patient welfare and a significant financial burden to health service providers, exemplified by estimated costs of up to ~£123 million per annum in the UK, and $424–451 million per annum in the USA (Jacobsen et al., 2008; Ploughman et al., 1999).

**Scheme 1:**
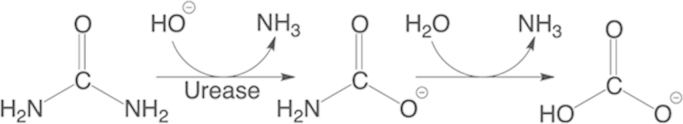
Urease-catalyserd hydrolysis of urea as a nitrogen source for *P. mirabilis*.

The problem of CAUTI is particularly pronounced in patients who are managed long-term with urethral catheterisation, where IUC are in place for weeks or months at a time. This includes many elderly individuals and those with spinal cord injuries, in whom urethral catheterisation is often used to manage incontinence in a community care setting (Buckley and Lapitan, 2009, Sørbye et al., 2005, McNulty et al., 2003, Getliffe, 1994). One of the most problematic and severe complications arising from CAUTI in this group is the encrustation and blockage of catheters, which may be experienced by up to 50% of patients undergoing long-term urethral catheterisation (Getliffe, 1994). Encrustation and blockage is almost exclusively due to infection by *Proteus mirabilis*, which is isolated from up to 45% of CAUTIs (Stickler, 2014, Stickler, 2008; Stickler et al., 1993; Mobley, 1996). Following the initial colonisation of the urinary tract, *P. mirabilis* develops extensive biofilm communities on catheter surfaces, characterised by aggregations of cells embedded in a dense exopolymeric matrix (Stickler 2014, 2008; Donlan, 2002). Biofilms are intrinsically resistant to immune clearance, antimicrobial agents, and environmental factors, hence treatment of infections involving biofilms is in itself a major medical problem (Stickler, 2014, Stickler, 2008; Donlan, 2002). Concurrent with biofilm development, *P. mirabilis* expresses a highly potent urease enzyme during growth in urine, allowing exploitation of urea as a nitrogen source (Griffith et al., 1976). The activity of this urease enzyme (Scheme 1) generates ammonia, elevating urinary pH, and leading to the precipitation of calcium phosphate and magnesium–ammonium phosphate from urine to form crystals of carbonate apatite [Ca_10_(PO_4_)_6_CO_3_], and struvite (MgNH_4_PO_4_·6H_2_O), respectively (Stickler et al., 1993; Griffith et al., 1976; Holling et al., 2014a, Holling et al., 2014b). These crystals subsequently become incorporated into the developing biofilms, which further stabilises and enhances their growth (Stickler et al., 1993; Holling et al., 2014a, Holling et al., 2014b). Through these processes, extensive abrasive crystalline biofilm structures are formed which encrust catheter surfaces and eventually block urine flow.

Catheter blockage causes painful retention of urine within the bladder, and subsequent vesico-ureteric reflux of infected urine to the kidneys (Stickler, 2014; 2008). If blockage is not detected before this occurs, patients suffer episodes of severe kidney infection and septicaemia (Stickler, 2014; 2008). Unfortunately, as the majority of long-term catheterised patients are cared for in the community, where constant clinical surveillance is not available, blockage typically remains unnoticed until life threatening consequences arise, and hospital treatment is required Stickler (2014). Although a range of catheters impregnated with antimicrobials are widely available, their use in controlling infection even during short-term catheterisation (<7 days) remains questionable (Pickard et al., 2012; Morris et al., 1997; Morgan et al. 2009).

Currently, two such “infection control” catheters are available in the United Kingdom National Health Service: a silver alloy-coated latex catheter, and a nitrofurazone-impregnated silicone plastic catheter. However, recent meta-analysis of clinical studies has shown that silver alloy-coated catheters were unable to significantly reduce symptomatic CAUTI compared with standard catheters, and were considered not to be cost-effective. Nitrofurazone-impregnated catheters showed borderline clinical benefit, however, any benefit was offset by a marked increase in patient discomfort, and concerns regarding the indiscriminate use of antibiotics (Pickard et al., 2012). Furthermore, all currently available catheter types remain susceptible to encrustation, including those with antimicrobial coatings (Stickler, 2014; Pickard et al., 2012; Morris et al., 1997; Morgan et al., 2009). As such, available infection control catheters provide little or no benefit for long-term catherised patients where the onset of bacteriuria (growth of bacteria in urine) is considered to be almost inevitable (Stickler, 2014; Jacobsen et al., 2008; Kunin, 1997). While bacteriuria and the majority of CAUTI are asymptomatic and typically not treated, the serious complications induced by blockage highlight the need for strategies to better identify and manage *P. mirabilis* infections, with the ultimate goal of warning patients and carers that blockage may be imminent (Stickler, 2014).

**Fig. 1:**
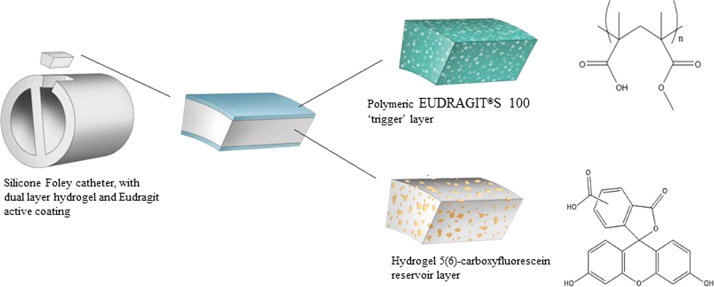
Schematic illustration of dual-layered polymeric architecture for pH-triggered release of 5(6)-carboxyfluorescein.

The concept of using urinary pH elevation to provide infection responsive drug release was explored by Irwin et al. (2013), who successfully achieved controlled release of nalidixic acid from poly(2-hydroxyethylmethacrylate (p(HEMA)) hydrogels. Here, a novel “early warning” system is described, which is designed to alert patients and carers of forthcoming catheter blockage. The system takes the form of an infection-responsive surface coating, compatible with existing catheter designs, able to provide a visual warning of *P. mirabilis* infection prior to encrustation and blockage. The coating consists of a dual-layered polymeric architecture, in which a lower layer of hydrogel (poly (vinyl-alcohol)) is employed to encapsulate the self-quenching dye 5(6)-carboxyfluorescein, at concentrations sufficient to inhibit fluorescence. This lower layer is capped and sealed by an upper pH-sensitive ‘trigger’ layer, ensuring no dye release while this is in place (Fig. 1). The ‘trigger’ layer is composed of EUDRAGIT®S 100 (an anionic co-polymer of methacrylic acid and methyl methacrylate). Elevation of urinary pH upon *P. mirabilis* infection (*via* the urease-catalysed hydrolysis of urea) causes dissolution of the upper EUDRAGIT®S 100 layer, releasing the carboxyfluorescein contained in the lower hydrogel matrix to provide a clear visual signal throughout the catheter drainage system that blockage is imminent, and intervention is required.

## 2 Experimental section

### 2.1 Silanisation of Foley catheters

In order to coat the hydrophobic silicone catheter with PVA, it was necessary to modify the surface with a hydrophilic moiety. In this case, a silane (3-Aminopropyl triethoxysilane) (APTES) introduced ethoxy groups on the catheter surface. The method for hydrophilisation of catheter surfaces via silanisation of the silicone surface was modified from Gencer et al. (2012). Catheters were washed in 1:1 mixture of ammonia (33% v/v) and hydrogen peroxide (30% v/v) for 10 min with constant shaking, then rinsed with deionized water and dried under nitrogen. The catheter was then placed in APTES (1% V/V) dissolved in dry N,N-Dimethylformamide (DMF) for 16 h. The surface-modified catheters were subsequently washed with DMF and dried under nitrogen. Water contact angle measurements were made to ensure the hydrophilic nature of the surface.

### 2.2 PVA hydrogel preparation

Poly(vinyl alcohol) (PVA,14,600–18,600 gmol^−1^) (20% w/v) was dissolved in deionized water and heated to 97 °C with constant stirring to facilitate dissolution. The cooled solution was stored at room temperature until required.

### 2.3 EUDRAGIT®S 100 solution preparation

EUDRAGIT®S 100 with a ratio of the free carboxyl groups to ester groups at approximately 1:2, and an average molecular weight of 150,000 g/mol was used*,* supplied directly from Evonik industries, Germnay. The organic dip coating solution of EUDRAGIT®S 100 was prepared according to the technical information (Evonik, 2015). EUDRAGIT®S 100 powder was added slowly to half of the diluent mixture, and stirred for 60 min until the polymer was completely dissolved. To the remaining diluent mixture was added talc and triethyl citrate, and the resultant suspension stirred for 10 min with a high shear mixer. The suspension was poured slowly into the EUDRAGIT®S 100 solution with constant stirring, before being Buchner filtered to remove the talc. The solution was stored at room temperature until required.

### 2.4 Coating of Foley catheters

To cooled PVA solution (*ca.* 20 °C) was added 5(6)-carboxyfluorescein (CF) solution (500 mM) at a 1:1 ratio. The resultant solution of hydrogel (10% w/v) containing CF dye (250 mM) was stored in the dark at room temperature. Catheters were coated with the gel/dye solution (100 µL) between the retention balloon and the tip, and stored at −20 °C overnight to promote cryogenic gelation. Catheters were thawed at room temperature (4 h) before dip coating with the pH-responsive trigger layer. Catheters were manually dip-coated 20 times in the EUDRAGIT®S 100 solution, with a 5 min solvent evaporation period at room temperature between each coating. Coated catheters were stored at 4 °C until required.

### 2.5 Artificial urine preparation

Sterile artificial urine was produced according to Stickler et al. (1999). A 5× concentrated stock solution was prepared containing anhydrous sodium sulphate (11.50 g/L), magnesium chloride hexahydrate (3.25 g/L), sodium chloride (23.00 g/L), tri-sodium citrate (3.25 g/L), sodium oxalate (0.10 g/L), potassium di-hydrogen orthophosphate (14.00 g/L), potassium chloride (8.00 g/L), ammonium chloride (5.00 g/L), gelatin (25.00 g/L) and tryptic soy broth (5.00 g/L), urea (125 g/L), and calcium chloride (3.25 g/L). Stock solutions of urea and calcium chloride were sterilised separately by membrane filtration (0.45 µm; Sartorius, United Kingdom) while other components were sterilised by autoclaving. For use in bladder models all components were combined and diluted to 1× strength using sterile deionised water, with the final pH adjusted to 6.1.

### 2.6 In vitro bladder models

Bladder model setup and operation was followed according to Stickler et al. (1999). The model consists of double-walled glass vessel maintained at 37 °C by an external water jacket (Fig. S1). The model was first sterilised by autoclaving, and coated catheters inserted aseptically into the vessel *via* silicone tubing attached to a glass outlet at the base. The catheter retention balloon was inflated using sterile water (10 mL) (achieving the sealing of the bladder outlet), and the catheter attached to a sterile drainage bag to form a full sterile closed drainage system. Sterile artificial urine was supplied to the bladder model *via* a peristaltic pump at a flow rate of 0.75 mL/min. This allows a reservoir (∼30 mL) of urine to collect in the bladder below the level of the catheter eyehole. As the volume of supplied urine increases, the overflow drains through the catheter to the collection bag. For bladder models simulating infection, residual urine in the models was inoculated directly with *P. mirabilis* or *E. coli* bacteria (10^10^ CFU). Clinical isolates of *P. mirabilis* RS1 were obtained from the Royal Sussex County Hospital, and uropathogenic *E. coli* NSM59 from University Hospitals, Bristol. Both strains were isolated from urinary tract infections. The bacterial cultures were allowed to incubate within the bladder model for 1 h to establish before flow was restored.

### 2.7 Quantification of viable bacterial cells in bladder models

Serial dilutions of the original bladder model culture were performed and plated on NSLB agar (tryptone (10 g/L), yeast extract (5 g/L), technical agar (20 g/L)). Each cell present will proliferate to form a visible colony that can be recorded and used to estimate the number of cells in the original culture, expressed as colony forming units/mL (CFU/mL).

## 3 Results and discussion

### 3.1 Infection models for evaluation of coating performance and catheter blockage

To evaluate the ability of the dual-layered “triggered-release” system to provide advanced warning of *P. mirabilis* infection and catheter blockage, the performance of coated catheters was tested using the *in vitro* bladder model system, originally described by Stickler et al. (1999) This model replicates a full sterile closed-drainage system, as used in clinical practice, providing a good representation of the *in vivo* catheterised urinary tract (Fig. S1). Prototype coatings were applied directly to the top 1 cm of silicone catheters, above the retention balloon (Bard all-silicone Foley catheters). Once the catheter is fitted, this coated region sits within the pool of residual urine that forms in the bladder. During infection, this sump of urine acts as a reservoir of bacteria and typically harbours a high bacterial load. The blockage of catheters can be readily identified in the bladder model system by monitoring the flow of urine.

Since changes to catheter surfaces may influence the rate of bacterial biofilm formation, it was first confirmed that coated catheters did not alter the ability of *P. mirabilis* to form crystalline biofilms and block catheters in this system. In models with coated catheters, *P. mirabilis* was able to rapidly encrust catheters and completely block urine flow in an average time of 16 h. This is comparable to the time taken for the same strain of *P. mirabilis* to block uncoated catheters under the same conditions, and no significant differences in time to blockage were evident between models fitted with coated or uncoated catheters (Fig. S2). In common with previous studies, (Holling et al., 2014a, Holling et al., 2014b, Holling et al., 2014a, Holling et al., 2014b) the encrustation and blockage of catheters in models infected with *P. mirabilis* was associated with an increase in urinary pH to an average of pH 8.55 on blockage. No significant differences were observed in the ability of *P. mirabilis* to elevate urinary pH or grow and persist in the system, in models fitted with coated catheters, compared to models fitted with standard uncoated catheters (Figure S2).

### 3.2 Activation of catheter coating and advanced warning of blockage

In order to evaluate the capacity of the triggered-release coating to provide advanced warning of catheter blockage, the release of dye and consequent colour change of urine during the course of bladder model experiments was monitored, and it was calculated how long before catheter blockage that this signal became apparent (duration of early warning). Since the application of this technology will be in alerting patients or their carers to the potential for impending blockage (requiring the signal to be easily visible without specialist equipment), these experiments were based on the direct subjective assessment of colour change by eye alone, under ambient lighting conditions. Observation of dye release was used to prompt associated quantitative analysis of urinary fluorescence, viable bacterial cell counts and pH of residual urine, with the blockage of catheters by *P. mirabilis* taken as the experimental end-point. In parallel, the response of coatings to *Escherichia coli* was also assessed, which is not only the most commonly encountered pathogen of the urinary tract overall, but also an example of a urease negative species unable to block or encrust catheters. Thus, models infected with *E. coli* served as additional controls to determine if coatings could discriminate urease producing *P. mirabilis* from non-urease producing species, which is vital for accurate early warning of blockage.

**Fig. 2:**
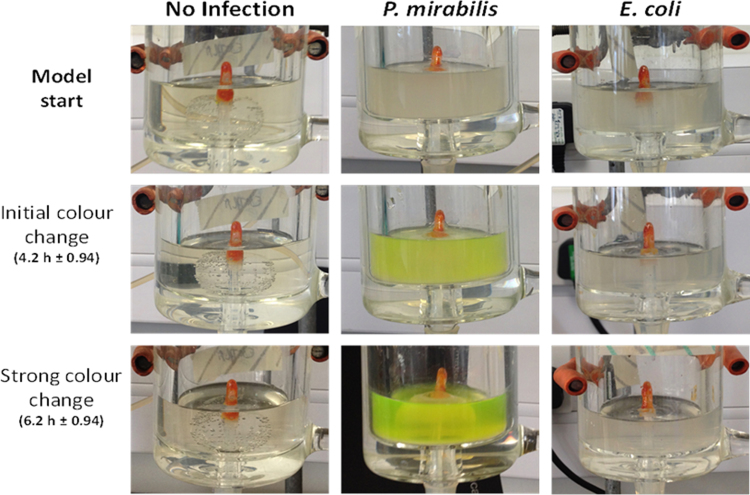
Example images documenting coating activation during the course of one experiment. *Columns* show example images taken from models infected with distinct bacterial species or devoid of bacteria: *No infection* – Un-inoculated controls devoid of bacteria. *P. mirabilis* – Models infected with a *P. mirabilis* clinical isolate. *E. coli* – Models infected with an *E. coli* clinical isolate (non-urease producing species). *Rows* show example images of bladder models at key time points related to observed coating activation: Model start – Images taken at the start of experiments. For Initial and Strong colour change figures in parentheses provide the average time after model start at which colour change was observed, and the standard error of the mean.

**Fig. 3:**
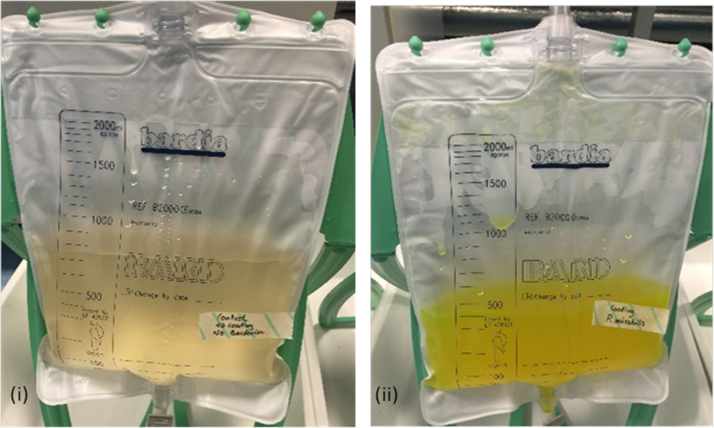
Visual colour change in urine collection bag in response to *P. mirabilis* infection. (i) Uninoculated standard catheter control (ii) *P. mirabilis* inoculated coated catheter control at 6.2 hours.

During the experiments, no obvious colour change was observed in models devoid of *P. mirabilis*. In uninfected models, or those infected with *E. coli,* coatings remained intact and no dye release visible to the naked eye was noted at any point throughout the experimental procedure (Fig. 2). This highlights the stability of the coatings in the absence of *P. mirabilis* infection. However, in models inoculated with *P. mirabilis*, a distinct colour change was observed in urine in bladder models an average of 4.2 h after the start of experiments. Although clearly visible in residual bladder urine, at the time of this colour change, the signal was not yet visible in urine collected in the drainage bag, and colour intensity continued to increase. Colour change in the urine appeared to reach a maximum intensity an average of 6.2 h after model activation. At this point, the colour change was clearly visible in urine accumulating in drainage bags (Fig. 3).

The intensity of the urine colouration reduced slightly over time, but was visible for the remainder of experiments, including within urine accumulated in drainage bags, until catheters became blocked at around 16 h on average. Removal of the Foley catheters from the control and *P. mirabilis* infected models 24 h after initial inoculation (8 h post-blockage), revealed that all encapsulated dye had been released from the coating in response to *P. mirabilis* infection, but not from the uninfected control models, which had remained intact despite constant contact with a flowing liquid culture.

**Fig. 4:**
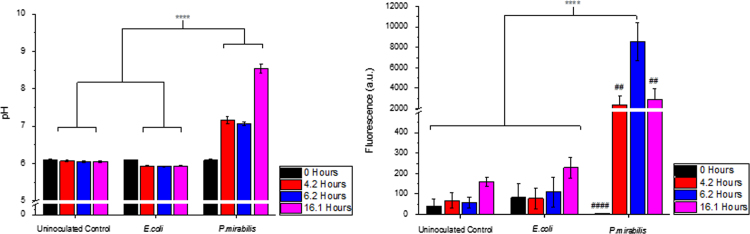
Analysis of *in vitro* bladder model conditions. (a) pH of residual bladder model urine at model start (0 h), on initial colour change (average 4.2 h), maximal colour change (average 6.2 h), and on catheter blockage (average 16.1 h) (b) Measured fluorescence of bladder model urine at the same time points. **** *p*<0.0001. Data shown is the mean of triplicate repeats. Error bars represent standard error of the mean (SEM). For part B only ## *p*<0.01; #### *p*<0.0001*P. mirabilis* at 0 h vs *P. mirabilis* at 6.2 h.

Dye release and changes in urine colour observed by eye were correlated with changes in pH of residual bladder urine in *P. mirabilis* infected models (Fig. 4a). A rise in urine pH of 1.2 pH units (from pH 6.1 to 7.3) was measured at the initial colour change (around 4 h) and was also evident at the point of strong colour change (around 6 h), increasing to a maximum of pH 8.6 at blockage. The measured fluorescence intensity of the bladder urine (Fig. 4b) in *P. mirabilis* infected models also corresponded with the observed dye release shown in Fig. 2. The two controls both exhibited negligible fluorescence intensity over all time points, suggesting that the coating was stable in artificial urine (pH 6.1) for the duration of these experiments. The *P. mirabilis* inoculated model, however, exhibited a large increase in fluorescence (ca. 20 fold) at the point of initial colour change (around 4.2 h) where pH had risen to 7.3. Fluorescence increased five times further when maximum colour change was observed (around 6.2 h), before decreasing somewhat by the time catheters had become blocked. The decrease between the maximum observed colour change at average of 6.2 h after model activation, and catheter blockage at around 16 h, would be due primarily to the dilution effects of continual urine flow and exhaustion of dye reservoir on the coated catheter.

Overall, when considered in the context of catheter blockage, the colour change induced by *P. mirabilis* activation of our prototype coatings provided ~10–12 h advanced warning of blockage, which is sufficient to permit intervention before the occurrence of any serious complications. A clear indication of *P. mirabilis* infection and imminent blockage being visible within accumulated urine in the drainage bags is also an important feature of the working system for several reasons: because this may be the only portion of the closed-drainage system that may be readily observed by patients or carers; and because drainage bags require regular emptying ensuring that this part of the system will be frequently checked.

Despite a lack of commercially available solutions for prevention or control of catheter blockage, the targeted detection of early *P. mirabilis* colonisation through monitoring of urinary pH has also been explored by others (Stickler et al., 2006a; Stickler et al., 2006b; Malic et al., 2012). These studies describe the creation of a pH sensor based on the incorporation of bromothymol blue into cellulose acetate or silicon polymers, which may be inserted as a connector between the drainage bag and catheter, or introduced into the drainage bag itself.(Stickler et al., 2006a; Stickler et al., 2006b; Malic et al., 2012) The performance of these sensors in early lab scale trials (also using the *in vitro* bladder model system) compares well to our surface coating and early warning of *P. mirabilis* induced blockage of between 12 and 43 h were reported in different tests (Stickler et al., 2006a; Malic et al., 2012).

Although in some cases a significantly greater duration of early warning was reported compared to our system, this almost certainly reflects the differences in the experimental setup, and the modelled infection scenario. In studies reporting a greater duration of early warning (43 h), models were inoculated with far fewer cells (~3 log lower) than used in our experiments, and were more reflective of earlier stages of infection (Stickler et al., 2006a). As such, overall time to blockage and elevation of urinary pH would be expected to take commensurately longer to achieve. However, examination of subsequent iterations of these sensors employed bladder model experiments more comparable to those conducted here, and provided early warning in a similar timeframe to the coating described in this work (Stickler et al., 2006a, Malic et al., 2012).

Overall, the coating system detailed here appears to perform as well as the bromothymol blue-based sensor approach, at least in terms of the *in vitro* bladder model system. However, the incorporation of this functionality into a surface coating applied directly to the catheter has numerous advantages over approaches that require the fabrication and use of additional components within the closed drainage system. The use of an additional component stands to not only increase the cost of providing this protection to patients, but also introduces additional complexity for patients and carers when fitting the system, as well as additional junctures at which the sterility of the system (the primary protection for the patient against infection) may be compromised. Some of these issues were highlighted in subsequent pilot scale clinical trials of these bromothymol blue based sensor devices that insert as connectors between catheter and drainage bag tubing (Long et al., 2014). In this trial some patients and carers reported issues with leakage, ease of use, and in a few cases that the extra tubing interfered with the ability to conceal their catheter drainage system (Long et al., 2014).

In contrast, these issues are all negated by the use of a surface coating approach. This strategy fits well with existing catheter manufacturing processes, where many variations of catheters with hydrogel or other surface coatings are already mass produced and commercially available. Further, the work here shows this is compatible with existing catheter designs. However, perhaps the principal advantage of the surface coating approach demonstrated here is the potential to add further functionality to these systems by incorporating additional active ingredients into the hydrogel reservoir. In this scenario, the triggered release and early visual warning of impending blockage could work in parallel with the release of antimicrobial agents, such as biocides or bacteriophage, to actively combat infection and slow or prevent blockage. The potential for this in comparable triggered release systems intended for application to prototype wound dressings has already been demonstrated, where virulence factor secretion by prominent wound pathogens can be used to trigger release of bacteriophage (Bean et al., 2014), and the enhancement of the catheter coatings in a similar fashion is also eminently feasible.

It is also important to consider the potential for activation of the coating by other uropathogens, and the implications of this for infection control. The lack of coating activity in models infected with *E. coli* indicates that our system can discriminate ureolytic pathogens from non-urease producers. Although *P. mirabilis* is the most common cause of catheter blockage, a number of other notable pathogens of the catherised urinary tract are urease producers and these include *Pseudomonas aeruginosa*, *Klebsiella pneumoniae*, *Providencia stuartii, Providencia rettgeri*, *Morganella morganii*, and *P. vulgaris* (Stickler, 2008; Broomfield et al., 2009). For the most part, urease activity in these other species is considerably less than that exhibited by *P. mirabilis*, and among these species only *Pr. retgerii, M. morganii*, and *P. vulgaris* have been reported to elevate urinary pH above pH 7, and to be capable of encrusting catheters (Broomfield et al., 2009). Because the coating is tuned to respond to changes in urinary pH, rather than the direct detection of urease activity or species specific factors, the coating should also be able to indicate infection by any species capable of sufficiently elevating urinary pH and causing blockage. This should permit the early warning of blockage and the need for intervention, regardless of the pathogen driving the elevation of urine pH.

The dissolution pH threshold of the trigger layer is also an important factor in the accuracy of this coating in signalling the potential for blockage. This requires a balance between providing an accurate signal of infection associated pH elevation, and providing a sufficient length of warning in patients where urinary pH may be more variable. Although uninfected urine typically has pH ranging from ~6.1–6.5, in some cases extremes of pH from a lower limit of 5.5 to an upper limit 7.0 have been reported (though these extremes are typically associated with other underlying disorders (Rose et al., 2015). However, perhaps the more critical value in terms of catheter blockage is the pH at which crystal formation is initiated, and this “nucleation” pH can also vary person to person and has been reported to range from pH 6.57–8.95 (Mathur et al., 2006). Given the range of values reported for urinary pH and nucleation pH, the current dissolution threshold of pH 7 would seem to offer a good compromise between the requirement for accurate warning and sufficient length of warning, and should be suitable for the vast majority of catheterised patients. This threshold is also congruent with levels of pH detected by previously reported bromothymol blue based pH sensors for monitoring *P. mirabilis* infection and warning of blockage (Stickler et al. 2006a; Malic et al., 2012). However, the optimisation of this aspect of coating function will need to be informed by larger scale evaluation in bladder models run with urine collected from human volunteers, and ultimately, clinical trials.

## 4 Conclusion

We have demonstrated a novel and previously unreported application of a pH-responsive polymer, in a dual-layered surface coating for urinary catheters. The coating was engineered to provide a visual response following a pH trigger: which correlates with an early warning of urinary catheter blockage as a result of *P. mirabilis* infection. This system provided a clear and unambiguous signal of infection throughout the catheter – urine collection bag closed drainage system, and duration of warning sufficient to permit intervention and thus potentially to prevent serious clinical complications. Performance was comparable to other reported sensor systems for early warning of blockage, which work on similar principles, although the implementation of this approach as a surface coating holds considerable manufacturing and practical advantages. Collectively, this work provides sound proof-of-concept for this infection-responsive surface technology and a firm foundation for its further optimisation and development, particularly in terms of increasing functionality through inclusion of antimicrobial agents. The diagnostic (and potentially theranostic) system described in this work has the potential to have a significant positive impact on patient welfare, and also reduce the cost of care, through prevention of catheter blockage and associated complications.

## Footnotes

Appendix A Supplementary data associated with this article can be found in the online version at doi:10.1016/j.bios.2016.02.059.
