# Supplementary material for: An in-situ infection detection sensor coating for urinary catheters
Source: Biosens Bioelectron. 2016 Jul 15;81:166–72. doi: 10.1016/j.bios.2016.02.059 (PMC4833687; doi:10.1016/j.bios.2016.02.059)
Supplement: Supplementary file 1 — Supplementary material [file mmc1.pdf]

Supporting Information

An *in-situ* infection detection sensor coating for urinary catheters

*Scarlet Milo, Naing Tun Thet, Dan Liu, Jonathan Nzakizwanayo, Brian V. Jones, A. Toby A. Jenkins**

S.E. Milo, N. T. Thet, D. Liu, A.T.A. Jenkins
Department of Chemistry, University of Bath, Bath, BA2 7AY, UK
E-mail: a.t.a.jenkins@bath.ac.uk

J. Nzakizwanayo, B.V. Jones
School of Pharmacy and Biomolecular Sciences, University of Brighton, BN2 4GJ, UK

B.V. Jones
Queen Victoria Hospital NHS Foundation Trust, East Grinstead, RH19 3DZ, UK

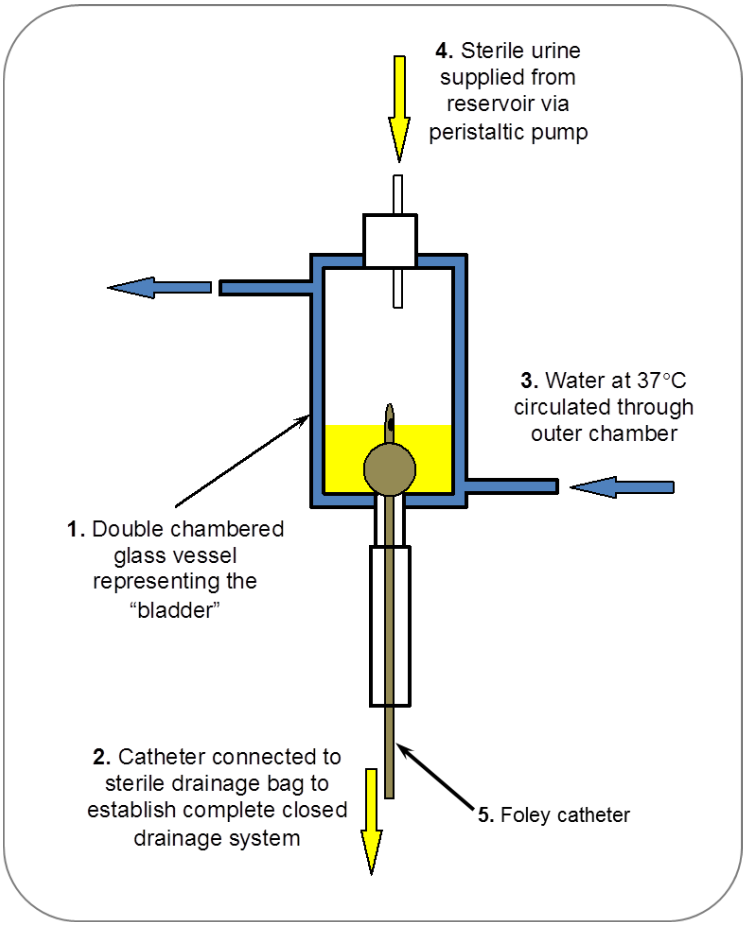

Supplementary figure 1. Diagram illustrating the in vitro bladder model system as originally described by Stickler et al 1999.

The potential for coating effects on ability of *P. mirabilis* for form crystalline biofilms, elevate urinary pH, and also cell viability was examined in the bladder model system (S figure 1). This confirmed that these parameters did not differ significantly between *P. mirabilis* infected models fitted with coated or uncoated catheters, during these experiments. Furthermore, no growth of bacteria was observed in non-inoculated models fitted with coated catheters demonstrating that the lab-scale aseptic manufacture of these modified catheters allowed production of sterile devices. Collectively, these initial evaluations confirmed that the prototype coating developed here can be robustly and meaningfully evaluated using the bladder model system.

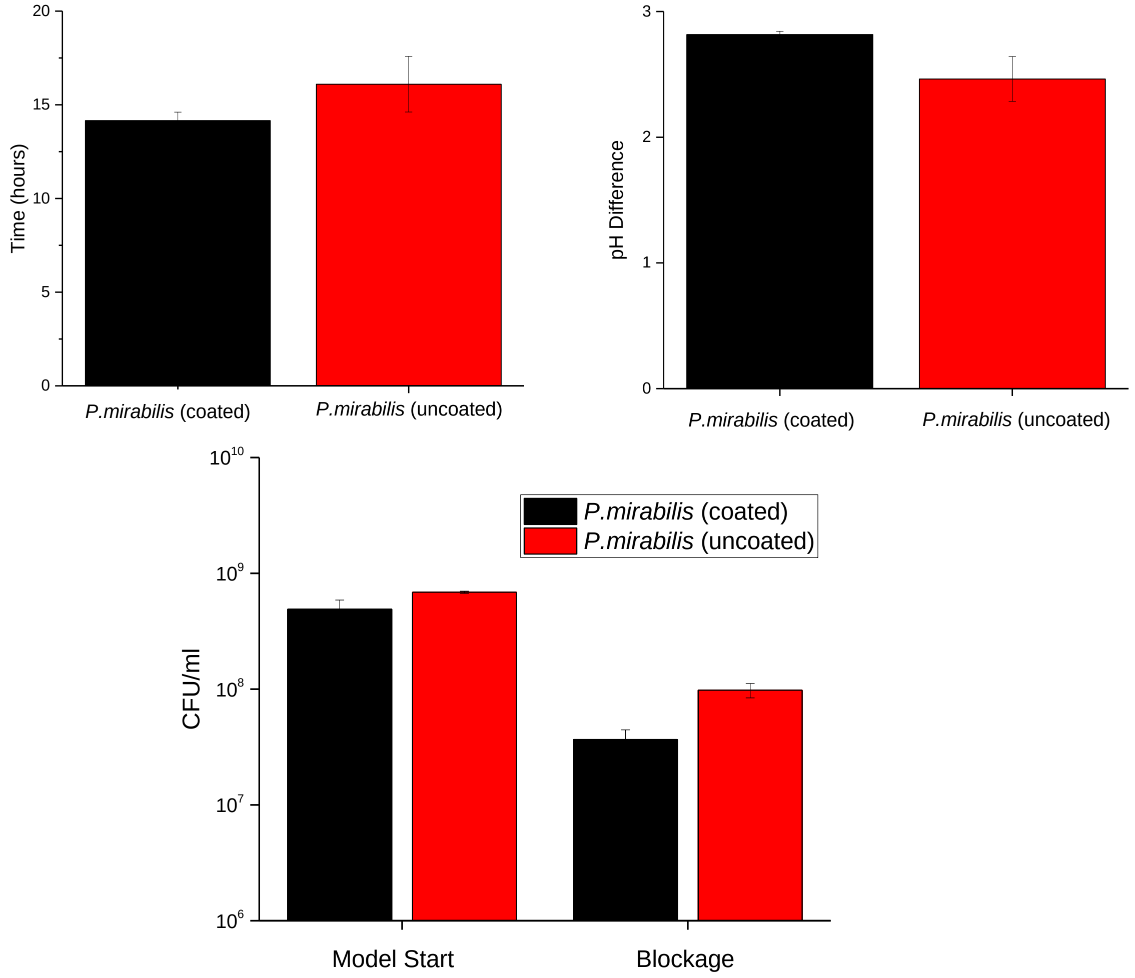

Supplementary figure 2. Assessment of coating impact on P. mirabilis behaviour in the bladder model system. A) Time taken for catheters **to** become blocked in models fitted with coated or uncoated all silicone catheters **B)** The rise in urine pH in *P. mirabilis* infected models fitted with coated or uncoated catheters. Baseline pH of the artificial urine media was 6.0-6.1, and data shows the number of pH units by which this rose between model activation and catheter blockage. **C)** Number of viable cells present in residual bladder model urine at the start of experiments and upon catheter blockage, in experiments with coated or uncoated catheters. All data represent the mean of three independent replicates. Error bars show SEM. No significant differences were indented in any data sets.

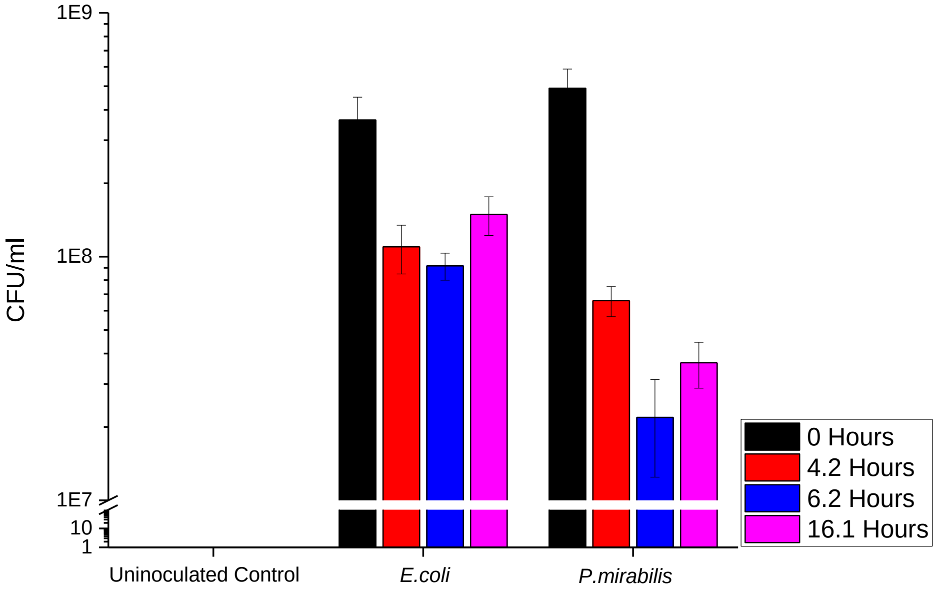

Supplementary figure 3. Enumeration of viable cells in models in residual "bladder" urine at key time points (no significant differences detected).
